# Repeatability assessment of sodium (^23^Na) MRI at 7.0 T in healthy human calf muscle and preliminary results on tissue sodium concentrations in subjects with Addison’s disease

**DOI:** 10.1186/s12891-022-05879-5

**Published:** 2022-10-20

**Authors:** Olgica Zaric, Hannes Beiglböck, Veronika Janacova, Pavol Szomolanyi, Peter Wolf, Michael Krebs, Siegfried Trattnig, Martin Krššák, Vladimir Juras

**Affiliations:** 1 High-Field MR Centre, Department of Biomedical Imaging and Image-Guided Therapy, Medical University of Vienna, Währinger Gürtel 18-20, 1090 Vienna, Austria; 2 Research Center for Medical Image Analysis and Artificial Intelligence (MIAAI), Department of Medicine, Faculty of Medicine and Dentistry, Danube Private University GmbH (DPU), Krems an der Donau, Austria; 3 Department of Medicine III (Division of Endocrinology and Metabolism), Medical University of Vienna, Vienna, Austria; 4 Department of Imaging Methods, Institute of Measurement Science, Slovak Academy of Sciences, Bratislava, Slovakia; 5 Christian Doppler Laboratory for Clinical Molecular MRI, Christian Doppler Forschungsgesellschaft, Vienna, Austria; 6 Institute for Clinical Molecular MRI in Musculoskeletal System, Karl Landsteiner Society, Vienna, Austria

**Keywords:** Sodium magnetic resonance imaging, Tissue sodium concentration, Calf muscle, Addison’s disease

## Abstract

**Objectives:**

To determine the relaxation times of the sodium nucleus, and to investigate the repeatability of quantitative, *in viv*o TSC measurements using sodium magnetic resonance imaging (^23^Na-MRI) in human skeletal muscle and explore the discriminatory value of the method by comparing TSCs between healthy subjects and patients with Addison’s disease.

**Materials and methods:**

In this prospective study, ten healthy subjects and five patients with Addison’s disease were involved. ^23^Na-MRI data sets were acquired using a density-adapted, three-dimensional radial projection reconstruction pulse sequence (DA-3DPR) with a modification for the relaxation times measurements. Differences in TSC between muscle groups and between healthy participants were analysed using a nonparametric Friedman ANOVA test. An interclass correlation coefficient (ICC) was used as the repeatability index. Wilcoxon rank sum test was used for evaluation of differences in TSC between study participants.

**Results:**

The mean T_1_ in the gastrocnemius medialis (GM), the tibialis anterior (TA), and the soleus (S) was 25.9 ± 2.0 ms, 27.6 ± 2.0 ms, and 28.2 ± 2.0 ms, respectively. The mean short component of T_2_^*^, T_2_^*^_short_ were GM: 3.6 ± 2.0 ms; TA: 3.2 ± 0.5 ms; and S: 3.0 ± 1.0 ms, and the mean long component of T_2_^*^, T_2_^*^_long_, were GM: 12.9 ± 0.9 ms; TA: 12.8 ± 0.7 ms; and S: 12.9 ± 2.0 ms, respectively. In healthy volunteers, TSC values in the GM were 19.9 ±0.1  mmol/L, 13.8 ±0.2 mmol/L in TA, and 12.6 ± 0.2 mmol/L in S, and were significantly different (*p* = 0.0005). The ICCs for GM, TA and S were 0.784, 0.818, 0.807, respectively. In patients with Addison’s disease, TSC in GC, TA, and S were 10.2 ± 1.0 mmol/L, 8.4 ± 0.6 mmol/L, and 7.2 ± 0.1 mmol/L, respectively.

**Conclusions:**

TSC quantification in a healthy subject’s calf at 7.0 T is reliable; the technique is able to distinguish sodium level differences between muscles and between healthy subjects and Addison’s disease patients.

**Supplementary Information:**

The online version contains supplementary material available at 10.1186/s12891-022-05879-5.

## Introduction

Over the last two decades, clinical research has demonstrated that biochemical information on disease progression and treatment efficacy may be obtained with a non-invasive metabolic imaging modality called sodium magnetic resonance imaging (^23^Na-MRI) [1]. The method enables quantification of tissue sodium concentration (TSC), which has been demonstrated to be a useful imaging marker for the investigation of different pathological conditions in almost every part of the human body [2]. Currently, the number of preclinical and clinical ^23^Na-MRI studies at 7.0 T is increasing, mainly because of the substantial gain in signal-to-noise ratio (SNR) at ultra-high fields [3–7]. An optimal SNR substantially improves image quality and reduces a patient’s scan time, thus confirming TSC as a marker for disease diagnosis and monitoring. However, an accurate sodium concentration quantification at ultra-high fields is challenging and depends on many factors, such as relaxation times (T_1_ and T_2_^*^) of the tissue of interest. Unfortunately, the knowledge about sodium relaxation times at 7.0 T of the muscle is still limited [8].

Sodium (^23^Na) is the primary cation in the human body and its concentration is connected to the physiological state of a cell and early changes in human homeostasis. Body hormonal imbalance or certain diseases, such as diabetes mellitus, hypertension, and acute heart failure, may cause an increase or a decrease of the sodium concentration in blood and manifest as conditions known as hypernatremia or hyponatremia [9, 10]. Changes of ^23^Na concentrations in the calf muscle, on the other hand, may be closely related to the molecular pathophysiology of the muscle tissue, which also may be common in rare diseases, such as muscle channelopathy [11] and dystrophy [5, 12–14]. Addison’s disease is another rare disorder characterized by impaired production of the steroid hormones cortisol and aldosterone, which may affect the sodium and potassium ion equilibrium (electrolyte imbalance) in the tissue [15]. Similar to the above-mentioned rare diseases, in subjects with Addison’s disease, sodium concentration level disturbances in a tissue may be potentially assessed using ^23^Na-MRI.

To be used as a reliable tool for different diagnostic and research investigations, this method requires an evaluation of repeatability and reproducibility, which should establish sodium tissue concentration assessments with sufficiently high accuracy.

The purpose of our study was to determine the relaxation properties of sodium nuclei in healthy calf muscle, and second, to perform reproducibility and repeatability tests of the sodium quantification measurements applicable in lower leg muscle investigations at 7.0 T. Further, we aimed to investigate whether regional differences in TSC of muscle are detectable and to explore the feasibility and discriminatory value of this method in patients with Addison’s disease.

## Materials and methods

All measurements were performed between July 2020 and June 2021 and were approved by the local research ethics committee. Written, informed consent was obtained from all participants in the study. All methods were performed in accordance with the Declaration of Helsinki.

### Hardware

MRI was performed on a whole-body 7.0 T MR scanner with a 70 mT/m gradient amplitude and a 200 mT/m/ms slew rate (Magnetom, Siemens Healthcare, Erlangen, Germany). Study participants were scanned in the supine, feet-first position. A dual-tuned sodium and proton knee coil (14 ^23^Na channels and a single ^1^H channel; Stark Contrast, Germany) was used.

### Phantoms

A cylindrical phantom made of poly methyl methacrylate (PMMA) filling completely the knee coil was filled with a mixture of agarose (4%) (Agarose, Sigma-Aldrich, USA) and a saline solution of 61.6 mmol/L, and was used for field mapping and corrections.

A set of calibration phantoms was made of 4% agarose and saline solution with four different sodium concentrations: 15.4, 30.8, 46.2, and 61.6 mmol/L, doped with 1% nickel sulfate. Concentration phantoms were attached to the inner side of the coil and were used for calibration curve derivation. To match the T_1_ value of the phantom and muscle tissue, we added 2.9 g/L copper sulfate to the mixture.

### Imaging protocol

All ^23^Na multi-channel data sets were acquired using a density-adapted, isotropic three-dimensional radial projection reconstruction pulse sequence (DA-3DPR) [16]. For T_1_ relaxation time measurements, a modified DA-3DPR with an inversion recovery (IR) pulse was included and five different inversion times (TI) were used. T_2_^*^ mapping was performed employing the same DA-3DPR sequence with 20 different echo times (TE).

A proton (^1^H) double echo steady state (DESS) sequence was acquired for partial volume effect (PVE) correction. Sodium imaging was performed using DA-3DPR followed by noise-only scans (no radio frequency [RF] power). More details on the sequences used can be found in Table 1.

**Table 1:** Imaging parameters of imaging sequences used in this study

Measurement	TR (ms)	TE (ms)	Matrix size	In-plane resolution (mm^3^)	FA (°)	Slice thickness (mm)	Inversion Time (ms)	Number of projections	Pulse duration (ms)	Acquisition time (min:s)
B_0_ mapping	139	0.60/1.60	128 × 128	4.0 × 4.0	90	4.0	/	4000	1.0	9:16
B_1_^+^ mapping	150	1.0	128 × 128	4.0 × 4.0	90	4.0	/	4000	2.0	10:00
T1 relaxation time	253–370	0.80	128 × 128	6.5 × 6.5	90	6.5	3.0–120	2600	1.5	64:03
T2* relaxation time	65	0.4–50.0	128 × 128	5.8 × 5.8	90	5.8	/	9000	0.68	48:45
Proton imaging	9.32	2.55	320 × 320	0.5 × 0.5	20	1.0	/	/	/	3:08
Sodium imaging	100	0.55	128 × 128	2.5 × 2.5	90	2.5	/	10,000	0.5	16:40

*B*_*0*_ external field, *B*_*1*_^+^ local transmitter field, *FA* Flip angle, *IT* Inversion time, *T1* longitudinal relaxation time, T2* Transverse “observed” relaxation time, *TE* echo time, *TR* repetition time

### Study participants

Ten healthy subjects were recruited. In four of ten participants, relaxation time measurements were performed. Repeatability tests were performed in all ten participants and planned in such a way that subjects underwent three lower leg muscle MRI examinations organized as two separate visits. Between the two scans (M1, M2), during one visit, the subject left the scanner for a five-minute-long break. Seven to eight days after the first visit, the same subject was scanned third time (M3). Volunteers were placed in a similar position according to anatomic landmarks from proton (^1^H) morphological imaging for the next scan. In addition, five participants with a diagnosis of Addison’s disease were scanned. Demographic data on study participants are shown in Table 2.

**Table 2:** Demographic data on participants involved in the study

**Subject number**	**Age**	**Gender**	**Diagnosis**	**BMI**	**Laterality**
1	27	Male	Healthy	19.7	Right
2	20	Male	Healthy	20.1	Right
3	26	Female	Healthy	21.9	Right
4	28	Female	Healthy	20.5	Right
5	32	Male	Healthy	22.5	Left
6	35	Male	Healthy	23.0	Left
7	34	Female	Healthy	20.8	Left
8	25	Female	Healthy	19.9	Left
9	36	Male	Healthy	21.0	Left
10	40	Female	Healthy	22.0	Right
11	33	Male	Addison’s disease	22.3	Right
12	30	Female	Addison’s disease	23.8	Right
13	49	Female	Addison’s disease	22.9	Right
14	54	Female	Addison’s disease	21.0	Right
15	40	Male	Addison’s disease	22.6	Right

Healthy subjects with no contraindications for MRI exam, such as cardiac implantable electronic devices, metallic intraocular foreign bodies, drug infusion pumps, implantable neurostimulation systems, cochlear/ear implant, drug infusion pumps, orthodontic braces, metallic particles, or items in or on the subject’s body, piercing and/or tattoo, and tendency towards claustrophobia were included in this study. The additional inclusion criteria for patients were that they had biochemically confirmed primary adrenal insufficiency and were on a stable hormone replacement therapy for at least three months prior the study according to routine clinical practice guidelines.

### Image reconstruction and inhomogeneity correction

Sodium images were reconstructed using an adaptive combining method (ADC) implemented in three dimensions to combine the complex images of the individual coils, with an interpolation factor of two and an overlap of eight pixels [17, 18]. SNR maps were calculated using a multiple replica approach [19]. The cylindrical homogeneous phantom which filled the knee coil and was used for B_0_ and B_1_^+^ maps generation, and it was scanned with the same geometry as subjects. The local transmitter B_1_^+^ field was mapped with a phase-sensitive method that applies a pulse combination of nominal flip angles of α_x_ = 180° about the x-axis and α_y_ = 90° about the y-axis [20]. External B_0_ field inhomogeneity correction was achieved by generating a data set with two different TEs; the first data set was acquired for off-resonance map calculations due to the resultant phase differences of two different echo times, and, in a second step, the data set was reconstructed multiple times with the corresponding off-resonance for each pixel to correct for B_0_ inhomogeneity. Sequence parameters are presented in Table 1. The correction factors derived from the cylindrical phantom were applied on *in vivo* data similar to previous work [21]. The feasibility of the applied method was evaluated on a set of agarose phantoms with different sodium concentrations comparing prepared sodium concentrations of calibration phantoms with the concentrations calculated after image corrections were applied [22] (supplementary material).

### Relaxation time calculations

T_1_ and T_2_^*^ relaxation time maps were derived by fitting the ^23^Na signal evolution to a mono-exponential function (T_1_) or a bi-exponential function (fast T_2_^*^ and slow T_2_^*^ components) using IDL software (version 6.3, Research Systems, Boulder, CO, USA) with mpfit function [23]. For T_1_ calculation five different TIs were used: 3, 15, 30, 60, 120 ms and for T_2_^*^ 20 different TEs were taken: 0.4, 0.6, 1.0, 2.0, 5.0, 10.0, 12.0, 14.0, 16.0, 18.0. 20.0, 22.0, 24.0, 26.0, 29.0, 32.0, 36.0, 40.0, 45.0 and 50.0 ms. For T_2_^*^, bi-exponential fitting procedure was performed on all MR data sets on a ROI-by-ROI basis ROI has been selected on the image acquired at the lowest TE (0.4 ms) and subsequently transferred to the rest of images. Mean sodium signal intensity has been stored for each ROI.

For bi-exponential fitting, a five-parametric function was used to fit the signal intensity:

1 $$SI={C}_{0s}{e}^{-\frac{TE}{{T}_{2s}^{*}}}+{C}_{0l}{e}^{-\frac{TE}{{T}_{2l}^{*}}}+offset$$

where T_2s_^*^ corresponds to the short component of T_2_^*^, T_2l_^*^ corresponds to the long component of T_2_^*^ C_0s_ and C_0l_ are the component ratios expressed further as a percentage value of C_0s_ + C_0l_: F_s_ = 100* C_0s_ /( C_0s_ + C_0l_) and F_l_ = 100* C_0l_ /( C_0s_ + C_0l_). Offset is again determined primarily by the noise in the image.

For T_1_ mapping, the following formula has been used:

2 $$SI=abs({M}_{m}\left(1-2{e}^{-\frac{TI}{{T}_{1}}}\right))+offset$$

where M_m_ is the maximum magnetization at full recovery, TI is the set of inversion times, and the offset reflects the noise.

### PVE corrections

Segmentation masks were created on morphological images acquired with DESS sequence with the parameters mentioned above and using a combination of automatic and manual segmentation (Functional Magnetic Resonance Imaging of the Brain (FMRIB) Software Library (v6.0, Analysis Group, FMRIB, Oxford, UK, the FMRIB's Automated Segmentation Tool (FAST) [24]) and ITK-SNAP, v3.8.0, Penn Image Computing and Science Laboratory (PICSL), University of Pennsylvania, Scientific Computing and Imaging Institute (SCI), University of Utah). Three different tissue compartments (delineated as regions of interests, ROIs) covering the certain muscle type (GM, S, TA) were used for PVE correction. The segmentation masks derived for three muscles and were co-registered with sodium images previously corrected for B_0_ and B_1_^+^ inhomogeneity. PVE calculation was performed according to a method proposed by Niesporek et al. [25], based on the geometric transfer matrix (GTM) method for PVE correction. The structural information was used in combination with a simulated point spread function (PSF) to calculate the corresponding region-spread functions (RSF) via convolution. For the PSF simulation and relaxation correction of the ^23^Na data sets, we used values that we obtained in healthy subjects.

### TSC assessments

Sodium data sets from every volunteer were reconstructed using an in-house-written MATLAB (R2020b, MathWorks Inc., Natick, MA) script. Values for ^23^Na-concentration in millimoles per liter (mmol/L) were calculated according to the formula (3) taking sodium signal intensities from selected ROIs and including them in the following formula:

3 $$\left[{Na}_{in\;vivo}\right]=\frac{I_{in\;vivo}^{corr}}{I_{phan}^{corr}}\left[{Na}_{ref}\right]$$

where $\left[{Na}_{in\;vivo}\right]and\left[{Na}_{ref}\right]$ are sodium concentrations in muscle of interest and reference concentration of 25 mmol/l [13, 26]. $I_{in\;vivo}^{corr}$ is signal intensity derived from ^23^Na images previously corrected for PVE, and B_0_ and B_1_^+^ inhomogeneity and ${I}_{phan}^{corr}$ is corrected signal derived form a calibration curve (supplementary material, Table 1). Additionally, $I_{in\;vivo}^{corr}$ and ${I}_{phan}^{corr}$ were corrected for relaxation time differences between muscle tissue and phantoms.

### Statistical analysis

Statistical analysis was done using IBM SPSS Statistics for Windows, version 22.0 (IBM, Armonk, NY). Metric data are described by means (mean ± SD) and normal distribution of measured values was tested using the Shapiro Wilks test. For categorical data, absolute frequencies and percentages are presented. Differences in TSC between the muscle regions were tested using a nonparametric Friedman ANOVA test. To investigate which pairs of muscles were significantly different in terms of TSC, we used a pairwise Wilcoxon signed-rank test with Bonferroni correction of *P*-values to compensate for multiple comparisons. Wilcoxon rank sum test was used for evaluation of differences in TSC between healthy volunteers and patients. A P value less than 0.05 was considered statistically significant.

The repeatability of measurements was estimated with an intra-class correlation coefficient (ICC) in test–retest analysis using a two-way mixed, absolute agreement model [27]. Based on the 95% confident interval of the ICC estimate, values less than 0.5, between 0.5 and 0.75, between 0.75 and 0.9, and greater than 0.90 were indicative of poor, moderate, good, and excellent repeatability, respectively [27]. Agreements between three measurements (M1, M2, M3) were assessed using Bland–Altman plots.

## Results

### Relaxation time measurements

**Fig. 1:**
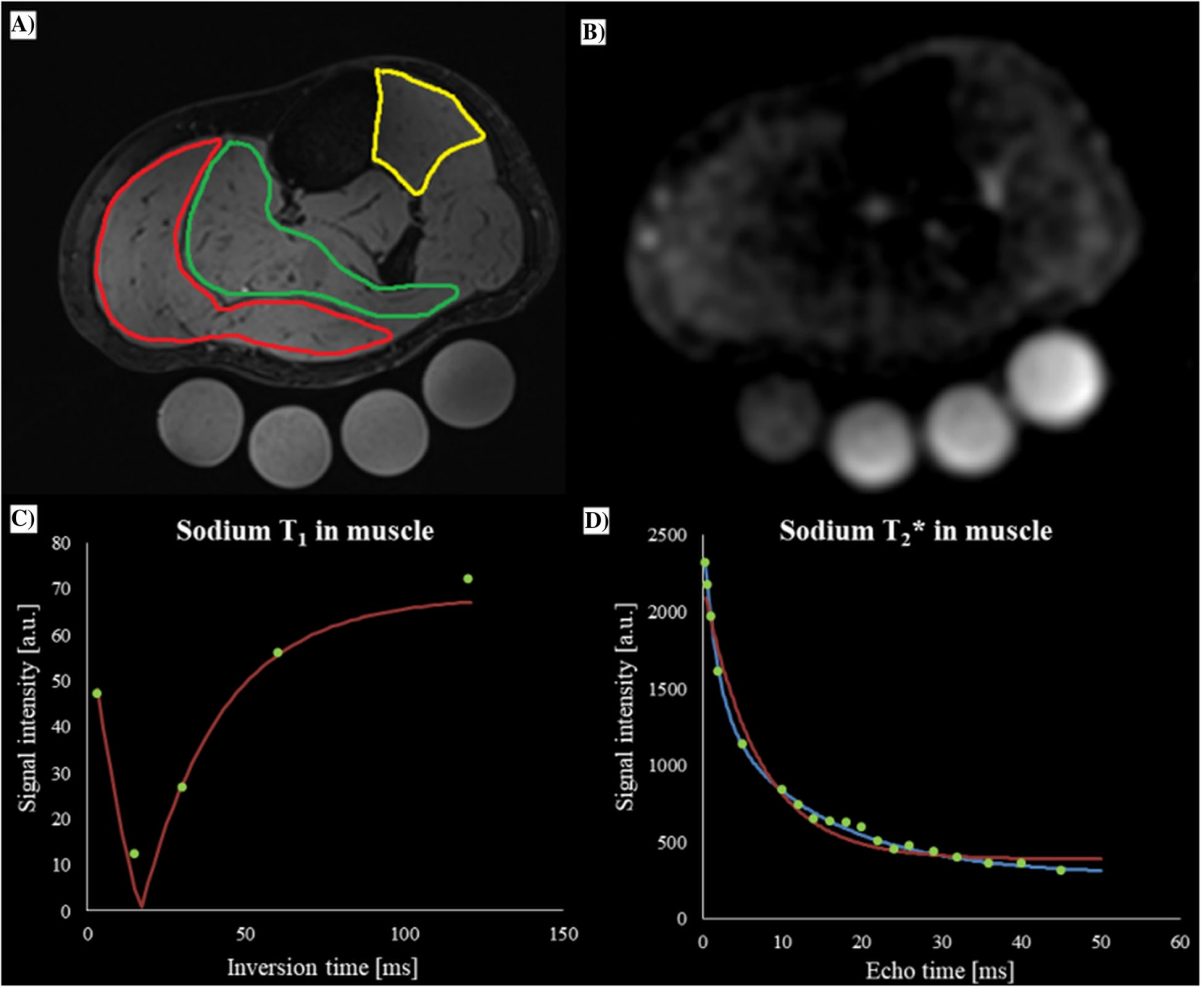
Lower calf images of the left leg of a 25-year-old healthy woman, in the axial orientation. **A** Proton (^1^H) DESS image showing three segmented regions of muscles that were used for analysis: gastrocnemius medialis (GM) (red); tibialis anterior (TA) (yellow); and soleus (S) (green). **B** Sodium (^23^Na) image of the same participant. **C** Graph shows measured inversion recovery data (green points), and T_1_ relaxation curve fit (red line), and **D** T_2_.^*^ relaxation time (measured data (green points); bi-exponential fit (blue curve), and mono-exponential fit (red curve)

In the subgroup of four healthy volunteers (age: 25 ± 4 years), the mean T_1_ and T_2_^*^ relaxation times were measured, calculated, and summarized in Table 3. The mean T_1_ in GM, TA, and S were: 25.9 ± 2.0 ms, 28.2 ± 2.0 ms, and 27.6 ± 2.0 ms, respectively. The mean short component of T_2_^*^, T_2_^*^_short_ were GM: 3.6 ± 2.0 ms; TA: 3.2 ± 0.5 ms; and S: 3.0 ± 1.0 ms, and the mean long component of T_2_^*^, T_2_^*^_long_, were GM: 12.9 ± 0.9 ms; TA: 12.8 ± 0.7 ms; and S: 12.9 ± 2.0 ms, respectively (Fig. 1).

**Table 3:** ^23^Na relaxation time values T_1_ [ms], T_2_^*^_short_ [ms], and T_2_^*^_long_ [ms] of the different regions of human calf muscle presented as means ± SD. In the brackets partition coefficient for two components of T_2_^*^ are given

**Subject number**	**Muscle type**	**T**_**1**_** [ms]**	**T**_2_^*^_long_** [ms]**	**T**_2_^*^_short_** [ms]**
1	gastrocnemius	28.5 ± 0.5	14.0 ± 0.5 (40)	3.4 ± 0.2 (60)
	tibialis anterior	29.4 ± 1.0	12.5 ± 0.3 (40)	3.1 ± 0.2 (60)
	soleus	28.9 ± 0.3	14.2 ± 0.3 (40)	2.5 ± 0.8 (60)
2	gastrocnemius	24.1 ± 2.0	12.6 ± 0.2 (40)	2.5 ± 0.3 (60)
	tibialis anterior	25.4 ± 0.3	13.7 ± 0.8 (40)	3.0 ± 0.4 (60)
	soleus	26.0 ± 0.2	12.7 ± 0.3 (55)	4.0 ± 3.0 (45)
3	gastrocnemius	24.4 ± 3.0	11.9 ± 0.2 (40)	2.7 ± 0.1 (60)
	tibialis anterior	26.4 ± 0.4	12.2 ± 0.2 (45)	2.6 ± 0.3 (55)
	soleus	27.7 ± 0.8	11.0 ± 0.2 (40)	2.6 ± 0.1 (60)
4	gastrocnemius	26.4 ± 0.9	13.0 ± 2.0 (40)	5.6 ± 0.2 (60)
	tibialis anterior	29.0 ± 2.0	12.7 ± 0.5 (40)	3.9 ± 2.0 (60)
	soleus	30.1 ± 0.9	13.4 ± 2.0 (45)	4.5 ± 0.6 (55)

### Repeatability tests

**Fig. 2:**
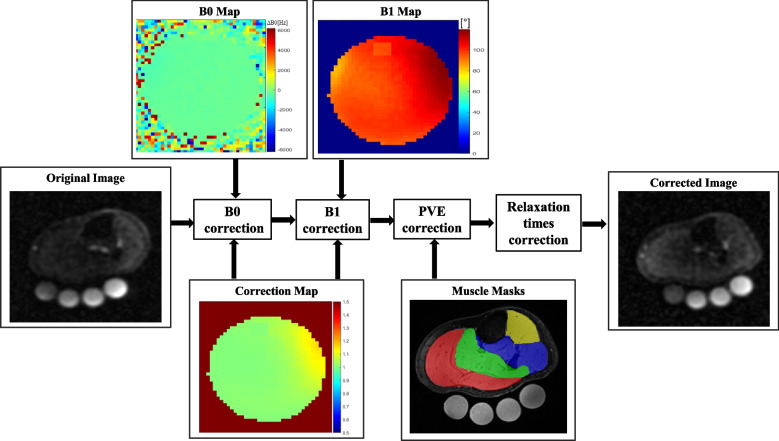
Flowchart diagram shows post-processing steps for in vivo ^23^Na-MRI data. The local transmitter B_1_^+^ field was mapped with a phase-sensitive method [20] and external B_0_ field inhomogeneity correction was achieved by generating a data set with two different echo times from ^23^Na data. B_0_ and B_1_^+^ measurements were performed on a cylindrical homogeneous phantom which filled the coil completely and was scanned under the same geometrical conditions as study participants. The correction factors derived from the phantom and were applied on in vivo data. The muscle segmentation mask for the reference compartments (red-gastrocnemius medialis, green-soleus, yellow-tibialis anterior, blue-other muscles) is created based a high-resolution ^1^H acquisition and co-registered to the ^23^Na images. The structural information was used in combination with a simulated point spread function (PSF) to calculate the corresponding region-spread functions (RSF) via convolution [25]. For the PSF simulation and relaxation correction of the ^23^Na data sets, we used values that we obtained in healthy subjects. Colored bars show distribution of calculated region-spread function values in arbitrary units (a.u.)

**Fig. 3:**
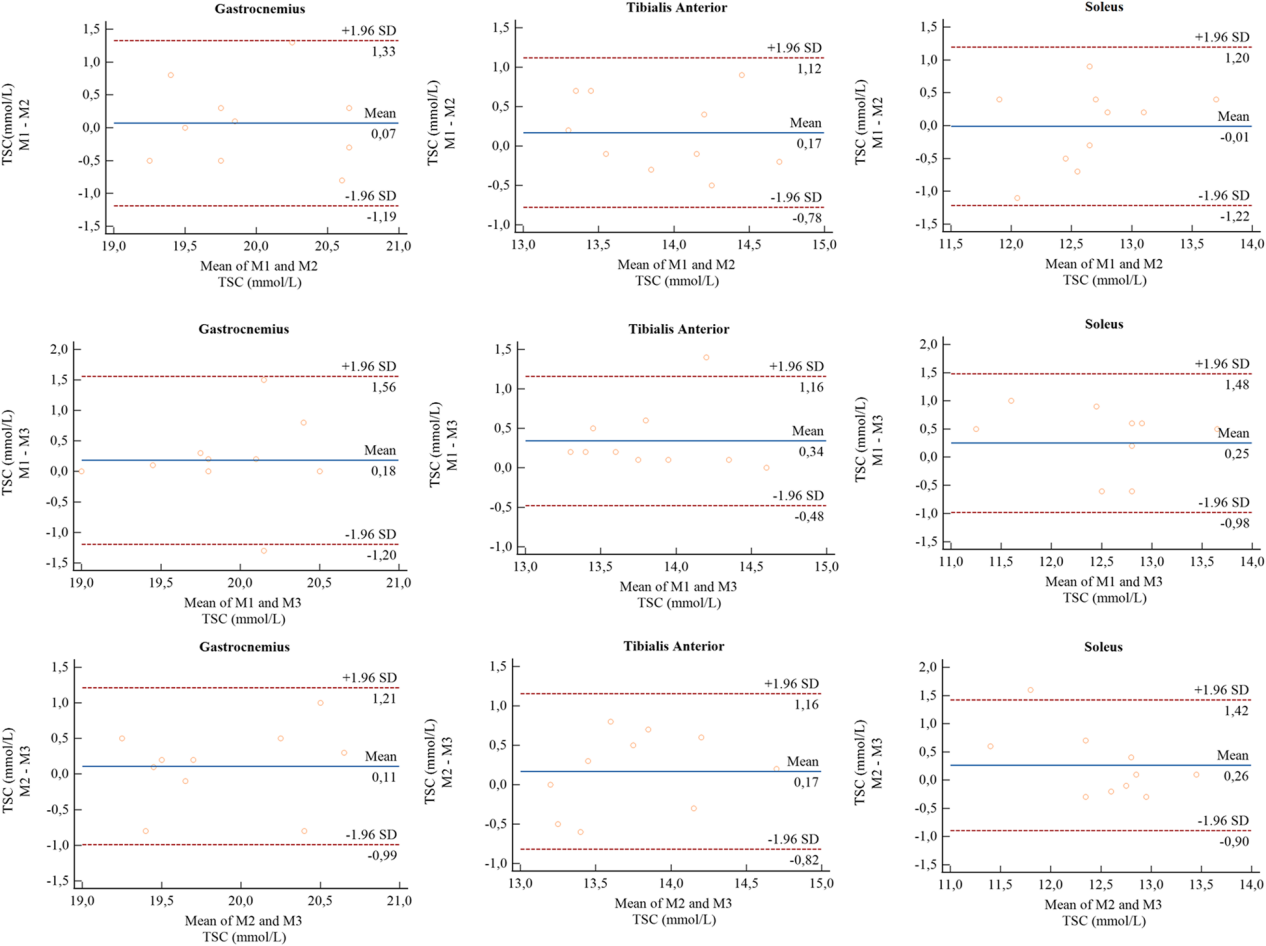
Bland–Altman (B-A) plots for three measurements (M1, M2, M3) for tissue sodium concentration (TSC) in three lower leg muscles: gastrocnemius medialis (GM); tibialis anterior (TA); and soleus (S) determined for healthy participants (*n* = 10). Bland–Altman plot was used to describe the agreement between two quantitative measurements. The horizontal axis of the plot represents the average of the two measurements and the vertical axis represents the difference between the two measurements. The mean value and lower and upper limits of the 95% confidence interval for the average difference are also present in the plot. Three B-A plots for each combination of measurements (M1 and M2, M1 and M2, M2 and M3) were constructed separately for each of the three muscles (gastrocnemius, tibialis anterior, and soleus). Each point represents one volunteer. In our case measurements are coded as follows: M1 – first MRI scan, M2 – MRI scan during the same session as M1 after a 5-min break and M3 – MRI scan performed 7–8 days after M1 and M2

Flowchart showing the image correction procedure is provided in Fig. 2. For the first measurement, the mean TSC values for GM, TA, and S were: 19.9 ± 0.5 mmol/L, 14.0 ± 0.5 mmol/L, and 12.7 ± 0.7 mmol/L, respectively. For the second scan, we found TSC of 19.9 ± 0.6 mmol/ for GM, 13.8 ± 0.6 mmol/L for TA, and 12.7 ± 0.5 mmol/L for S. The third exam for GM, TA, and S found TSC values of 19.8 ± 0.5 mmol/L, 13.7 ± 0.5, mmol/L and 12.4 ± 0.8 mmol/L, respectively. ICC test–retest analysis showed a good repeatability for each group of muscles individually. The ICC for the gastrocnemius medialis was 0.784 (CI 95%, lower bound = 0.468, upper bound = 0.951), for the tibialis anterior, the ICC was 0.818 (CI 95%, lower bound = 0.435, upper bound = 0.948), and, for the soleus, the ICC was 0.807 (CI 95%, lower bound = 0.435, upper bound = 0.948). Bland–Altman diagrams of reproducibility tests show that majority of points are scattered between the limits of agreement, except for the comparison of M1 with M3 in tibialis anterior, M2 with M3 in tibialis anterior, and M2 with M3 soleus muscle. There is no apparent pattern in the Bland–Altman plot – the difference between measurements does not change as the average of measurements increases and indicates a good agreement between the three measurements performed in the same subject (Fig. 3).

### TSC quantification of the calf muscles

Ten healthy subjects, five men and five women (age: 30 ± 6 years, body mass index (BMI), 21.1 ± 1.0 kg/m^2^) were involved in repeatability tests. The mean SNR of sodium images of the healthy human calf was 16 ± 3. In subjects with Addison’s disease the mean SNR was 13 ± 5. Before corrections were applied, the mean TSC values for GM, TA, and S were 22.7 ± 0.7 mmmol/L, 17.8 ± 1.0 mmol/L, and 16.4 ± 0.9 mmol/L and, respectively. After all corrections were applied, the mean TSC of three measurements were: gastrocnemius medialis: 19.9 ± 0.1 mmol/L; tibialis anterior: 13.8 ± 0.2 mmol/L; and soleus: 12.6 ± 0.2 mmol/L (Table 4).

**Table 4:** Tissue sodium concentration (TSC) determined after external (B_0_) and local (B_1_) field inhomogeneity corrections and partial volume effect (PVE) in healthy subjects in different muscle regions and during three imaging sessions (measurements), M1, M2, and M3

Subject number↓ Muscle type →	TSC [mmol/L]—M1			TSC [mmol/L]—M2			TSC [mmol/L]—M3		
	**GM**	**TA**	**S**	**GM**	**TA**	**S**	**GM**	**TA**	**S**
1	20.2 ± 0.8	13.4 ± 0.7	12.2 ± 0.8	21.0 ± 0.8	13.2 ± 0.2	12.7 ± 0.3	20.0 ± 0.3	13.2 ± 0.2	12.8 ± 0.3
2	20.5 ± 0.9	13.7 ± 0.9	12.9 ± 0.9	20.8 ± 0.4	14.0 ± 0.8	12.5 ± 0.2	20.5 ± 1.0	13.2 ± 1.0	12.7 ± 1.0
3	19.9 ± 0.2	14.6 ± 1.0	13.2 ± 0.4	19.8 ± 0.6	14.8 ± 1.0	13.0 ± 1.0	19.6 ± 2.0	14.6 ± 0.8	12.6 ± 0.7
4	20.8 ± 0.5	14.0 ± 0.4	12.5 ± 0.9	20.5 ± 1.0	14.5 ± 0.6	12.8 ± 0.9	20.0 ± 0.8	13.9 ± 0.9	13.1 ± 0.7
5	20.0 ± 0.8	14.9 ± 0.2	13.1 ± 1.0	19.6 ± 0.8	14.0 ± 0.5	12.2 ± 0.2	19.4 ± 1.0	13.5 ± 0.5	12.5 ± 0.2
6	19.5 ± 1.0	13.8 ± 1.0	13.9 ± 0.5	20.0 ± 2.0	13.1 ± 0.1	13.5 ± 0.5	20.8 ± 0.9	13.7 ± 0.7	13.4 ± 0.4
7	19.9 ± 0.9	13.5 ± 0.5	12.9 ± 1.0	19.6 ± 1.0	13.6 ± 0.6	12.7 ± 0.7	19.7 ± 0.6	13.3 ± 0.6	12.0 ± 0.5
8	19.5 ± 0.8	13.7 ± 1.0	12.1 ± 0.4	19.5 ± 0.9	13.0 ± 0.7	11.7 ± 0.8	19.4 ± 2.0	13.5 ± 0.1	11.1 ± 0.3
9	19.0 ± 0.2	14.1 ± 0.7	11.5 ± 0.8	19.5 ± 1.0	14.2 ± 0.6	12.6 ± 0.2	19.0 ± 0.5	13.5 ± 0.4	11.0 ± 0.6
10	19.8 ± 0.5	14.4 ± 0.8	12.2 ± 0.2	19.0 ± 0.7	14.0 ± 0.2	12.9 ± 1.0	19.8 ± 0.9	14.3 ± 1.0	12.8 ± 1.0
11^a^	9.6 ± 0.6	7.7 ± 0.7	7.2 ± 0.8						
12^a^	10.7 ± 0.8	8.0 ± 0.8	7.1 ± 1.0						
13^a^	11.1 ± 0.8	9.0 ± 1.0	7.1 ± 2.0						
14^a^	10.8 ± 0.9	8.8 ± 2.0	7.4 ± 0.5						
15^a^	8.8 ± 1.0	8.7 ± 0.9	7.3 ± 0.4						

*G* Gastrocnemius, *M1, M2, M3* the first session, the second, and the third imaging session, respectively, *S* Soleus, *TA* Tibialis anterior, *TSC* Tissue sodium concentration

^a^Addison’s patient

Nonparametric Friedman test showed a significant difference between TSC values for all three types of muscles (*P* = 0.0005). There was a difference between gastrocnemius medialis and soleus muscle (*P* = 0.018) and between gastrocnemius medialis and tibialis anterior (*P* = 0.018). A difference between the soleus and tibialis was found as well (*P* = 0.018).

In participants with Addison’s disease (age: 41 ± 10 years, BMI: 22.5 ± 1.0 kg/m^2^) the mean TSC measured in the gastrocnemius medialis was 10.2 ± 1.0 mmol/L. In the tibialis anterior and soleus, measured TSC values were 8.4 ± 0.6 mmol/L and 7.2 ± 0.1 mmol/L, respectively (Table 4).

**Fig. 4:**
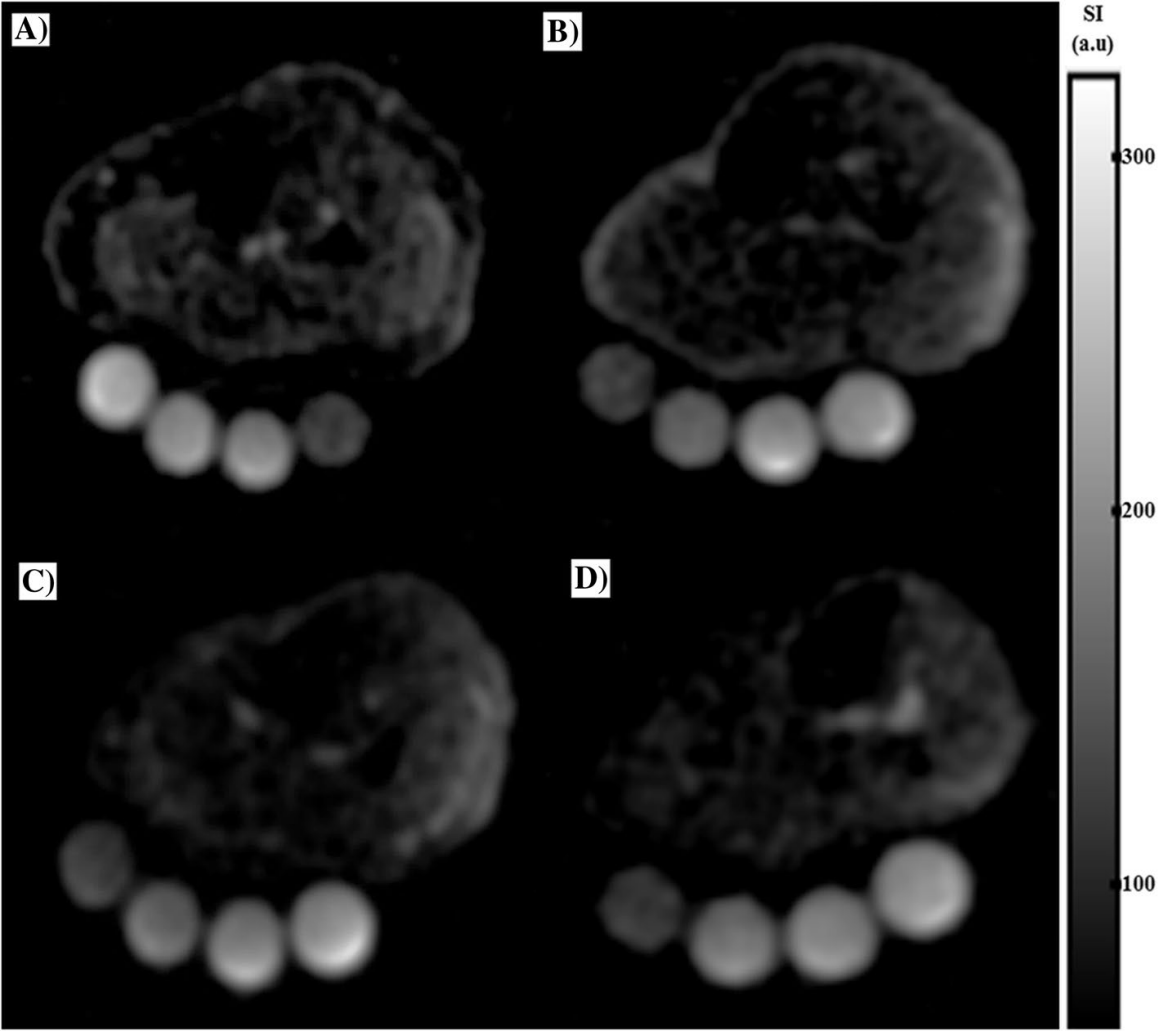
Sodium magnetic resonance imaging (^23^Na-MRI) of lower calf of the right leg. ^23^Na-MRI in the transversal orientation of (**A**) a 41-year-old healthy woman, and (**B**) in a 32-year-old healthy man. ^23^Na-MRI in the axial orientation of (**C**) a 49-year-old woman (**D**) and a 33-year-old man with a diagnosis of Addison’s disease; gray scale bar shows image signal intensity (SI) in arbitrary units (a.u.)

A significant difference in TSC values was found between healthy volunteers and patients for all three muscles: *P* = 0.0007, *P* = 0.0027 and *P* = 0.0007 for GM, TA and S, respectively (Fig. 4).

## Discussion

Tissue sodium concentration can be considered a useful parameter for biochemical investigations of muscular pathologies. At present, TSC values reported in the literature indicate substantial differences between individual reports of this parameter assessed in human calf muscle. This is most probably due to the lack of established MRI quantification protocols available and different quantification methodologies applied. The utilization of ultra-high system such as 7 T enabled investigation of many different relevant clinical questions in relatively short scanning times, sodium imaging with substantially higher SNR and higher resolution compared to 3 T. This is particularly important for investigation of i.e., endocrinological disorders by quantifying sodium concentrations in skin and muscle as organs where disease can be non-invasively diagnosed and monitored.

Our study was performed at 7.0 T MRI and involved ten healthy subjects and included relaxation time measurements, TSC quantification, and repeatability tests. Relaxation time measurements demonstrated no substantial differences in T_1_, T^*^_2long_, and T^*^_2short_ of three different muscle types. Sodium concentration quantification in healthy participants showed significant differences in TSCs in three different parts of the calf muscle (gastrocnemius medialis, tibialis anterior, and soleus) (*P* = 0.0005). In healthy participants, repeatability tests showed good repeatability of sodium concentration quantification in three different regions of the calf muscle (ICC = 0.784–0.948). The Bland–Altman showed no apparent pattern in the plot – the difference between measurements does not change as the average of measurements increases. The points on the Bland–Altman plot were uniformly scattered between the limits of agreement, therefore, we could conclude that there was a good agreement between every two pairs of measurements.

In addition, we examined and quantified TSCs in the calf muscle of five subjects with a diagnosis of Addison’s disease and found substantially lower TSC values compared to those in healthy participants. This finding is in line with our expectation and the choice of AGS as a model for ^23^Na^+^ imbalances. Glucocorticoids and mineralocorticoids play a major role in the regulation of sodium balance. Interestingly, these differences could be observed despite adequate hormone substitution therapy in patients with adrenal insufficiency. Whether ^23^Na-MRI might play a useful role in the clinical evaluation of adequate treatment should be evaluated in future trials.

The relaxation T_1_ times of ^23^Na of the healthy human lower leg muscle at 3.0 T reported in the literature were about 30 ms [26, 28]. Measurements of relaxation times of the ^23^Na nucleus of lower leg muscle measured at 7.0 T showed values that were slightly different from those obtained on 3.0 T scanners. The short and long components of T_2_^*^ at 3.0 T were reported to be 0.7 ± 0.1 ms and 13.2 ± 0.2 ms, respectively [29]. In our study, T_2short_^*^ component was higher, most probably due to limitation of imaging sequence and minimum echo time of 0.4 ms that could be used.

Moreover, because of different physiological and pathological contributions (edema, volume regulation, etc.) to the sodium concentration, the ^23^Na-MRI measurements in patients may be potentially confounded by T_1_ and T_2_^*^ relaxation effects, and consequently, the values can actually deviate from the values measured in healthy tissue [14].

Quantitative TSC values of the lower leg muscle determined noninvasively with ^23^Na-MRI in healthy male subjects are in good agreement with the results obtained invasively by chemical analysis in patients who require extremity amputation [30]. TSC values reported in the literature are in the range of 13.0 to 25.0 mmol/L [31]. Gast et al. recently reported TSC after B_0_ corrections for the gastrocnemius medialis, assessed at two sites, as 17.0 ± 2.2 mmol/L for site ≠ 1 and 16.2 ± 1.3 mmol/L for site ≠ 2. For the tibialis anterior, TSC at two sites were 14.3 ± 1.3 mmol/L and 13.7 ± 1.2 mmol/L, respectively. For the soleus, TSC values were 18.1 ± 1.4 mmol/L and 17.5 ± 1.0 mmol/L, respectively [3], confirming significant differences in TSC between GM, TA, and S muscle (*p* < 0.0005). Recently, Ahlulail et al. reported substantially higher TSC values after using T_2_* corrections and found the highest concentrations of sodium in the soleus muscle (34.1 ± 2.2 mmol/L) compared to GM (25.0 ± 2.8 mmol/L) and TA (25.3 ± 2.1 mmol/L) [29]. This discrepancy in the highest sodium content found in different muscles reported in the literature need to be further investigated using standardized protocols and applied on a larger group of subjects.

The difference in TSC values before and after corrections were from 12 to 23% depending on muscle type. Since that we have good homogeneity of external and local fields, we assume that this discrepancy mainly originates from PVE correction. The largest contribution of PV correction (34 ± 1) % to the total correction factor was found by Lott et al. who has measured TSC in human heart. In calf muscle tissue of the healthy subjects, Lott et al. found TSC of 20 ± 3 mM at 7.0 T which is in a good agreement with our results [28]. The influence of PVE correction on TSC differences between muscle types should be further investigated.

Repeatability investigations of ^23^Na-MRI in lower leg [4, 29, 31] or in upper leg muscle [32] have been already reported in the literature; however, measurement conditions for different research centers may be different (i.e. external magnetic field strength, coils, etc.), and repeatability may vary from one to another investigation site. Therefore, we performed our own tests to demonstrate the repeatability of quantification methodology applied in this study. Our study has some limitations. First, our population group was too small to have an appropriate analysis of sex- and age-related differences in TSC. Sodium relaxation times in Addison’s and healthy subjects muscle tissue may be different as well. Moreover, it is known that ^23^Na concentration in the calf of women can be hormonally dependent, which makes standardization of quantification more challenging [33]. Second, our healthy participants were slightly younger than the Addison’s disease patients. Future studies should include age matching and obese subjects who may have lower TSC values in calf muscle, most probably due to a fat infiltration. In this study, BMIs between the participants were comparable, but it would be interesting to investigate possible TSC differences between obese adults and patients Addison’s disease, as TSC differences between them may have different origin [34].

The post-processing procedure is time-consuming, and further development of the image reconstruction methods is necessary. However, we performed evaluations in the post-processing phase, so they didn’t influence the participant’s scanning time. In favor of reduced workload for the clinicians, we didn’t perform segmentation of calibration tubes and blood vessels, which may influence PVE corrections due to the high Na^+^ concentration in blood and phantoms. Similarly, the muscle segmentation we performed on three slices instead of the whole calf volume could influence TSC quantification accuracy and should be the subject of future research.

A short acquisition time is extremely important if measurements must be performed on patients to reduce potential motion artefacts and keep discomfort to a minimum. To investigate further connection of sodium relaxation time and different pathological conditions, the application of sodium magnetic resonance fingerprinting (^23^Na-MRF) would enable much faster data acquisition for relaxation time measurements [35, 36]. Relaxometry, in this context, may have a crucial role in disease detection and efficacy evaluation of different treatment approaches.

## Conclusion

Sodium concentrations in the tissue may be reliably assessed using ^23^Na-MRI at 7.0 T. The technique is quantitative and sensitive enough to distinguish differences in sodium levels between heathy and pathological tissue. Early metabolic changes in the tissue may be evaluated with ^23^Na-MRI. This might provide information on the effectiveness of glucocorticoid and mineralocorticoid replacement therapy in adrenal insufficiency and could be a useful parameter with which to evaluate ongoing treatment.

## Supplementary Information

**Additional file 1. **Supplementary material.

## Data Availability

The datasets generated and/or analysed during the current study are not publicly available due their size and complexity but are available from the corresponding author on reasonable request.

## Abbreviations

AC: Adaptive combine
AUC: Area under the curve
*B*_0_: Static magnetic field
*B*_1_: Combined transmit and receive radiofrequency field
B_1_^+^: Transmit radiofrequency field
GC: Gastrocnemius medialis
ROI: Region-of-interest
S: Soleus
TA: Tibialis anterior
TSC: Tissue sodium concentration
23Na-MRI: Sodium magnetic resonance imaging

## References

[1] Madelin G (2014). Sodium MRI: methods and applications. Prog Nucl Magn Reson Spectrosc.

[2] Zaric O (2020). Frontiers of sodium MRI revisited: from cartilage to brain imaging. J Magn Reson Imaging.

[3] Gast LV (2021). Combined imaging of potassium and sodium in human skeletal muscle tissue at 7 T. Magn Reson Med.

[4] Gerhalter T (2020). Assessing the variability of (23) Na MRI in skeletal muscle tissue: reproducibility and repeatability of tissue sodium concentration measurements in the lower leg at 3 T. NMR Biomed.

[5] Gerhalter T (2020). Quantitative (1)H and (23)Na muscle MRI in Facioscapulohumeral muscular dystrophy patients. J Neurol..

[6] Utzschneider M (2021). Towards accelerated quantitative sodium MRI at 7 T in the skeletal muscle: comparison of anisotropic acquisition- and compressed sensing techniques. Magn Reson Imaging.

[7] Utzschneider M (2020). Accelerated quantification of tissue sodium concentration in skeletal muscle tissue: quantitative capability of dictionary learning compressed sensing. MAGMA.

[8] Madelin G, Jerschow A, Regatte RR (2012). Sodium relaxation times in the knee joint in vivo at 7T. NMR Biomed.

[9] Kopp C (2013). 23Na magnetic resonance imaging-determined tissue sodium in healthy subjects and hypertensive patients. Hypertension.

[10] Hammon M (2015). 23Na Magnetic resonance imaging of the lower leg of acute heart failure patients during diuretic treatment. PLoS ONE.

[11] Weber MA (2016). 7-T (35)Cl and (23)Na MR imaging for detection of mutation-dependent alterations in muscular edema and fat fraction with sodium and chloride concentrations in muscular periodic paralyses. Radiology.

[12] Weber MA (2011). Sodium (23Na) MRI detects elevated muscular sodium concentration in Duchenne muscular dystrophy. Neurology.

[13] Gerhalter T (2019). (23) Na MRI depicts early changes in ion homeostasis in skeletal muscle tissue of patients with duchenne muscular dystrophy. J Magn Reson Imaging.

[14] Kushnir T (1997). In vivo 23Na NMR studies of myotonic dystrophy. Magn Reson Med.

[15] Liamis G, Milionis HJ, Elisaf M (2011). Endocrine disorders: causes of hyponatremia not to neglect. Ann Med.

[16] Nagel AM (2009). Sodium MRI using a density-adapted 3D radial acquisition technique. Magn Reson Med.

[17] Walsh DO, Gmitro AF, Marcellin MW (2000). Adaptive reconstruction of phased array MR imagery. Magn Reson Med.

[18] Benkhedah N (2016). Evaluation of adaptive combination of 30-channel head receive coil array data in 23Na MR imaging. Magn Reson Med.

[19] Minarikova L.e.a. Adaptive combine reconstruction of sodium MRI data of breast and knee at 7 T: optimization and comparison to sum-of-square reconstruction. Proc Intl Soc Mag Reson Med. 2017;25(2017). Abstract #5226

[20] Morrell GR (2008). A phase-sensitive method of flip angle mapping. Magn Reson Med.

[21] Zaric O (2021). Tissue sodium concentration quantification at 7.0-T MRI as an early marker for chemotherapy response in breast cancer: a feasibility study. Radiology.

[22] Zaric O (2016). Quantitative sodium MR imaging at 7 T: initial results and comparison with diffusion-weighted imaging in patients with breast tumors. Radiology.

[23] Available from: https://www.l3harrisgeospatial.com/docs/mpfit.html.

[24] Jenkinson M (2002). Improved optimization for the robust and accurate linear registration and motion correction of brain images. Neuroimage.

[25] Niesporek SC (2015). Partial volume correction for in vivo (23)Na-MRI data of the human brain. Neuroimage.

[26] Nagel AM (2011). 3 Tesla sodium inversion recovery magnetic resonance imaging allows for improved visualization of intracellular sodium content changes in muscular channelopathies. Invest Radiol.

[27] Koo TK, Li MY (2016). A Guideline of Selecting and Reporting Intraclass Correlation Coefficients for Reliability Research. J Chiropr Med.

[28] Lott J (2019). Corrections of myocardial tissue sodium concentration measurements in human cardiac (23) Na MRI at 7 Tesla. Magn Reson Med.

[29] Alhulail AA (2021). Fast in vivo (23) Na imaging and T 2 * mapping using accelerated 2D-FID UTE magnetic resonance spectroscopic imaging at 3 T: proof of concept and reliability study. Magn Reson Med.

[30] Kopp C (2012). (23)Na magnetic resonance imaging of tissue sodium. Hypertension.

[31] Dyke JP (2018). Reliability and agreement of sodium ((23)Na) MRI in calf muscle and skin of healthy subjects from the US. Clin Imaging.

[32] Milani B (2019). Exploring a new method for quantitative sodium MRI in the human upper leg with a surface coil and symmetrically arranged reference phantoms. Quant Imaging Med Surg.

[33] Wang P (2017). Sex differences in sodium deposition in human muscle and skin. Magn Reson Imaging.

[34] Roth S (2019). Tissue sodium content and arterial hypertension in obese adolescents. J Clin Med.

[35] Kratzer FJ (2020). Sodium relaxometry using (23) Na MR fingerprinting: a proof of concept. Magn Reson Med.

[36] Kratzer FJ (2021). 3D sodium ((23) Na) magnetic resonance fingerprinting for time-efficient relaxometric mapping. Magn Reson Med..

