# Supplementary material for: Repeatability assessment of sodium (23Na) MRI at 7.0 T in healthy human calf muscle and preliminary results on tissue sodium concentrations in subjects with Addison’s disease
Source: BMC Musculoskelet Disord. 2022 Oct 20;23:925. doi: 10.1186/s12891-022-05879-5 (PMC9585786; doi:10.1186/s12891-022-05879-5)
Supplement: Supplementary file 1 — Additional file 1. Supplementary material. [file 12891_2022_5879_MOESM1_ESM.docx]

**Supplementary material**

The first calibration curve was generated by plotting the prepared phantom sodium concentrations (from 15.4 mmol/L to 61.6 mmol/L) versus the original signal intensities values (I^orig^_phan_) measured on sodium images. After we corrected the signal intensities of the phantoms (I^corr^_phan_), we re-read from the curve new (calculated) phantom sodium concentrations (Table 1).

The second calibration curve was generated by plotting calculated phantom sodium concentrations versus corrected signal intensities and from this curve, we read the corrected signal (I^corr^_phan_) that corresponds to the reference concentration [N_a_*_ref_*] of 25.0 mmol/L and this value was replaced in formula 3 (main document). In this formula, I^corr^_invivo_ stands for corrected sodium signal intensity derived from the muscle of interest.

**Table 1**: Sodium concentrations of prepared phantoms and recalibrated phantom sodium concentrations derived from the standard curve. The mean calculated phantom sodium concentration ± standard deviations (SD) are given as average from different measurements

| Phantom No. | Prepared phantom concentration (mmol/L) | Calculated phantom concentration (mmol/L) |
| --- | --- | --- |
| 1 | 15.4 | 14.5±0.8 |
| 2 | 30.8 | 29.8±1.0 |
| 3 | 44.6 | 42.2±0.6 |
| 4 | 66.4 | 64.3±0.7 |
